# A magnetic nanoscale metal–organic framework (MNMOF) as a viable fluorescence quencher material for ssDNA and for the detection of mercury ions *via* a novel quenching–quenching mechanism†

**DOI:** 10.1039/c9ra08274c

**Published:** 2020-01-22

**Authors:** Muppidathi Marieeswaran, Perumal Panneerselvam

**Affiliations:** Department of Chemistry, SRM Institute of Science and Technology Kattankulathur 603 203 Tamil Nadu India panneerp1@srmist.edu.in panneerchem82@gmail.com +91 9688538842

## Abstract

A novel fluorescent biosensor has been designed and synthesized comprising a magnetic nanoscale metal–organic framework (MNMOF) functionalized with fluorescein amidite (FAM)-labeled ssDNA. It exhibits good sensitivity and selectivity for Hg(ii) cations over other co-existing metal ions. MNMOF was fabricated by a one-pot synthetic method and it was successfully characterized with various techniques such as UV-visible spectroscopy, fluorescence spectroscopy, Fourier-transform infrared (FT-IR) spectrometry, X-ray diffraction (XRD), scanning electron microscopy (SEM), transmission electron microscopy (TEM) and X-ray photoelectron spectroscopy (XPS). The FAM-labeled ssDNA was adsorbed onto the surface of MNMOF through π–π stacking and electrostatic interactions, which resulted in the partial quenching of its fluorescence intensity (65%). Upon the subsequent addition of Hg(ii) ions, the fluorescence intensity was further quenched at 52%, due to the re-adsorption of dsDNA onto the surface of MNMOF. Thus, the FAM-labeled ssDNA showed a drastic decrease in fluorescence intensity with Hg(ii). This quenching–quenching mechanism led to a linear response in the fluorescence intensity to Hg(ii) concentration (*R*^2^ = 0.934) with a low detection limit of 8 nM. The specific merits of MNMOF make it an ideal platform for mercury sensor applications.

## Abstract 2

Here, we propose a magnetic nanoscale metal–organic framework as a novel quenching material and reveal its quenching–quenching mechanism. It sensitively and selectively binds Hg(ii) and a detection limit of 8 nM.

## Graphical abstract

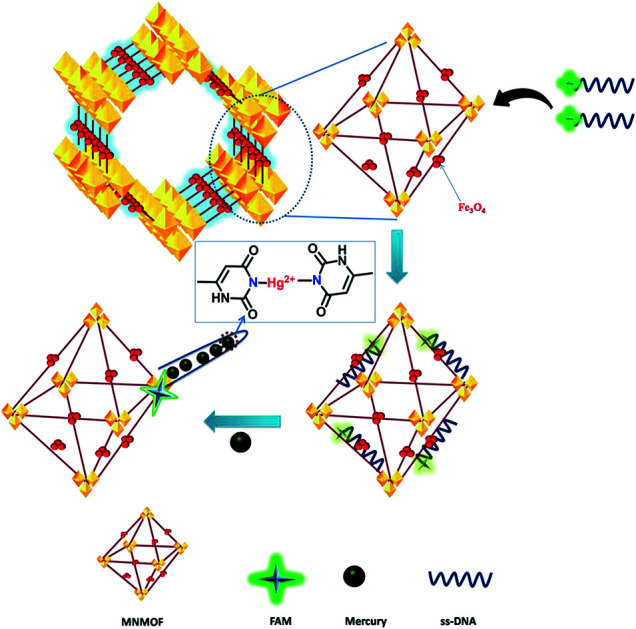

## Introduction

The development of biosensors based on highly sensitive fluorescent probes for the selective detection of heavy metals and transition metal ions is difficult, but it has been an area of focus for the scientific community in the past few years due to concerns regarding human health and environmental safety. Mercury ions have been considered as one of the most toxic heavy metals, causing Minamata diseases, neurodevelopment disorders, subclinical brain dysfunction, kidney failure, and cancer. Furthermore, the World Health Organization (WHO) has set a permitted limit for Hg(ii) in drinking water of 30 nM. Therefore, environmental monitoring of aqueous Hg(ii) is in increasing demand.^1,2^

So far, many analytical methods have been proposed for the detection of Hg(ii) such as atomic absorption spectroscopy, inductively coupled plasma mass/atomic emission spectrometry, ultraviolet (UV)-visible spectroscopy, high-performance liquid chromatography, colorimetric and electrochemical sensors, and so on.^3–8^ The existing techniques have limitations: they can be time-consuming, and require high-cost instruments and highly skilled personnel. To overcome the drawbacks, nucleic acid-based fluorescent sensors have been proposed.^9–12^ Among the existing techniques, fluorescence-based nucleic acid (T–Hg(ii)–T) sensors have been described using various dyes such as fluorescein amidite (FAM), tetramethylrhodamine (TAMRA), and Cys-labeled oligonucleotides for the precise detection of mercury. Recently, numerous fluorescence quencher materials such as graphene oxide (GO), nanoparticles (NPs), carbon nanotubes (CNTs), MoS_2_ and polydopamine nanotubes^13–18^ have been used more frequently for the detection of mercury ions.

The progressive development of synthetic processes for the production of metal–organic frameworks (MOFs) has been reported as well as their unique characteristics linked to fascinating properties such as excellent performance-selectable composition, high surface area, tunable porosity, high electrical conductivity, and optical properties.^19–22^ As expected, nanoscale metal–organic frameworks (NMOFs) have promising properties, such as high functionality, strong bio-affinity, and high stability. NMOFs have shown potential applications in versatile fields, such as catalysis, drug delivery, adsorption, gas storage, photocatalysis, and sensing applications.^24–27^ Moreover, various synthetic routes have been employed to produce NMOFs, such as nanoscale precipitation, and solvothermal, surfactant-template, and reverse microemulsion methods.^23^

Very recently, some NMOFs have been designed for detection based on the effective fluorescence sensing platform of small molecules and DNA. Studies have been documented with MOFs including UiO-66-NH_2_, MIL-88B, H2dtoaCu, and MIL-101, which show tremendous abilities to bind with dye-labeled DNA, quenching its fluorescence intensity.^28–30^ NMOFs bind to dye-labeled ssDNA through electrostatic, π–π stacking, and/or hydrogen-bonding interactions. Therefore, the fluorescence is quenched by photoinduced electron transfer (PET) and fluorescence resonance energy transfer (FRET) mechanisms.^31^

Fe_3_O_4_ NPs have received great attention because of their magnetic and adsorption properties.^32^ Yuling and co-workers fabricated Fe_3_O_4_@MOFs for photocatalytic contaminant removal and drug delivery.^33^ Yan and co-workers synthesized Fe_3_O_4_@MOF for analytical applications *via* magnetic solid-phase extraction.^34^ Zhang and co-workers designed and synthesized Fe_3_O_4_@MOF (MIL-53(Fe)) for the degradation of organic pollutants.^35^

In this work, we focused our efforts on FAM-labeled ssDNA adsorbed on the surface of a magnetic NMOF (MNMOF)-based biosensing platform to increase the sensitivity for Hg(ii) detection. The FAM-labeled ssDNA could be adsorbed on the surface of MNMOF through π–π stacking interactions, which led to a quenching of the fluorescence emission to 65% of the initial level. The addition of Hg(ii) further enhanced the quenching of the fluorescence at 52%, as a result of Fe^3+^ inorganic metal nodes present on the surface of MNMOF. To the best of our knowledge, this is the first report of a fluorescence quenching–quenching mechanism based on an MNMOF for an environmental application. The strategy gave a good analytical response in the determination of mercury. Furthermore, this MNMOF has a high quenching ability compared to those of other nano quencher materials.

## Methods

### Materials and measurements

All chemicals were purchased from Sigma Aldrich, Avra and SRL chemicals and were of analytical grade. These included ferric chloride (FeCl_3_), 2-aminoterephthalic acid, dimethylformamide (DMF), methanol, mercury nitrate, cadmium chloride, ferric chloride, magnesium chloride, silver chloride and other standards. Solvents were used without any further purification. Aqueous solutions were prepared from Milli-Q water. The buffer was prepared from 50 mM Tris–HCl, NaCl (50 mM) and MgCl_2_ (5 mM) (pH = 7.4). The stock solution of DNA was prepared by using Tris–HCl buffer. The synthesized DNA was purchased from Integrated DNA Technologies (IDT, Avantor, India) and the DNA probe sequence was 5′-56-FAM-ATT TGT TTT GTT TCC CCT TTC TTC TTT TCT TTT-3′

Fluorescence spectroscopy was performed on a HORIBA scientific spectrofluorometer using cuvettes of 1.0 cm path length with a xenon lamp excitation source and an excitation wavelength of 490 nm. The morphology and crystalline structure of MNMOF were evaluated using scanning electron microscopy (SEM, FEI Quanta FEG 200) and high-resolution transmission electron microscopy (HR-TEM, JEOL, JEM, Fb-2000). X-ray diffraction (XRD) was performed using a PAN analytical X'pert pro X-ray diffractometer with CuKα radiation. The bonding information studies were performed on an Agilent Technologies FTIR spectrometer (USA); Fourier transform infrared (FT-IR) spectra were recorded in attenuated total reflectance (ATR) mode.

### Synthesis of NMOF NH_2_-MIL-101(Fe)

NH_2_-MIL-101(Fe) was prepared using a previously reported method with some modifications.^36^ Typically, 1.6 g of FeCl_3_ and 1.4 g of 2-aminoterephthalic acid were dissolved in DMF/methanol with stirring at ambient temperature. The resulting homogenous solution was transferred to a 100 mL Teflon-lined autoclave and then heated at 150 °C for 12 h. After cooling down to room temperature, the brown precipitate was separated by centrifugation, washed with DMF and methanol and dried in a hot-air oven at 60 °C overnight.

### Synthesis of Fe_3_O_4_ nanoparticles

Fe_3_O_4_ NPs were prepared using a simple hydrothermal technique based on the previously reported literature.^21^ In brief, 5 mmol of FeCl_3_·6H_2_O and 43.8 mmol of CH_3_COONa were dissolved in 40 mL of ethylene glycol and magnetically stirred for 45 minutes. The obtained solution was transferred into a 100 mL Teflon-lined stainless-steel autoclave and kept in a muffle furnace at 200 °C for 10 h. After cooling to room temperature, the obtained black precipitate was separated, washed several times with distilled water and ethanol and finally dried in ambient conditions.

### Synthesis of MNMOF

MNMOF was synthesized *via* a one-pot method. Fe_3_O_4_ nanospheres (0.020 g), FeCl_3_·6H_2_O (1 mmol) and 2-aminoterephthalic acid (1 mmol) were added into 15 mL of DMF solution and ultrasonicated for about 15 min. The mixture was then transferred into a pressure tube with a volume capacity of 60 mL and heated at 130 °C for 2 days. After the heat treatment, the product was collected by centrifugation at 6000 rpm. To remove excess solvent and unreacted precursor, the obtained powder was dried under vacuum at 60 °C for 12 h.

### Analysis of environmental water samples

The sample tap water was collected from our laboratory. This environmental sample was filtered through a membrane to remove any insoluble impurities. Aliquots of the environmental water sample were spiked with a stock solution of Hg(ii) and diluted with Tris–HCl buffer. Then fluorescence measurements were performed.

## Results and discussion

**Fig. 1:**
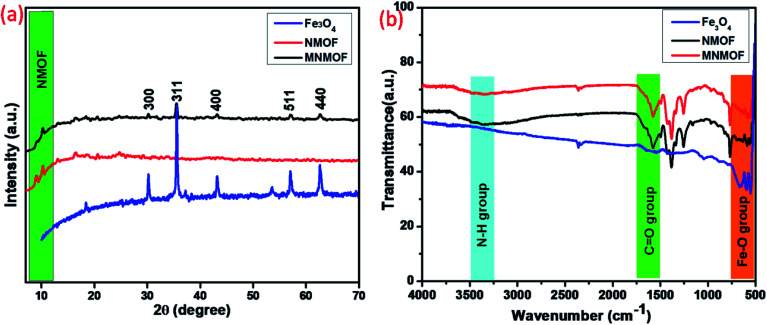
(a) PXRD patterns of Fe_3_O_4_ NPs, NMOF and MNMOF, (b) FT-IR spectra of Fe_3_O_4_ NPs, NMOF and MNMOF.

Structural information from powder X-ray diffraction (PXRD) studies of the synthesized materials, namely Fe_3_O_4_ NPs, NH_2_-MIL-101(Fe) (NMOF), and NH_2_-MIL-101(Fe)@Fe_3_O_4_ (MNMOF), are represented in Fig. 1a. The NMOF characteristic diffraction peaks exist at low 2*θ* values of 9.04°, 10.30°, and 13.10°, consistent with previous literature reports. Furthermore, the formation of Fe_3_O_4_ NPs is demonstrated from the crystalline nature and diffraction peaks at 30.2°, 35.5°, 43.1°, 57.1°, and 62.7° corresponding to the (220), (311), (200), (511), and 400, planes of Fe_3_O_4_, respectively.^37^ Finally, we confirmed the formation of MNMOF from the combination of Fe_3_O_4_ NPs and NMOF diffraction peaks found at 10.30°, 30.2°, 35.5°, 43.1°, 57.1°, and 62.7°. These data indicate that the Fe_3_O_4_ NPs are incorporated to give MNMOF without any disturbance to the NMOF crystalline nature. Moreover, MNMOF does not show any detectable crystalline impurities or peak shifts indicating its high purity. Fig. 1b illustrates the FT-IR spectra of Fe_3_O_4_ NPs, NMOF and MNMOF to clarify the chemical bonding interactions. As displayed in Fig. 1b, the FT-IR spectrum of NMOF shows two major functional groups, namely amine (N–H) and carbonyl (C=O), through sharp absorption peaks at 3477 cm^−1^ (N–H str), and 1679 cm^−1^ (C=O) respectively. The Fe_3_O_4_ NPs have strong peaks at around 669 cm^−1^, corresponding to the Fe–O band. Moreover, the MNMOF spectrum shows a combination of the peaks of the parent materials, with a broad peak at 3477 cm^−1^ attributed to the stretching frequency of N–H, and peaks at 1568 and 1398 cm^−1^ ascribed to the symmetric and asymmetric frequency of the carbonyl group. These FT-IR results confirm the formation of MNMOF.

**Fig. 2:**
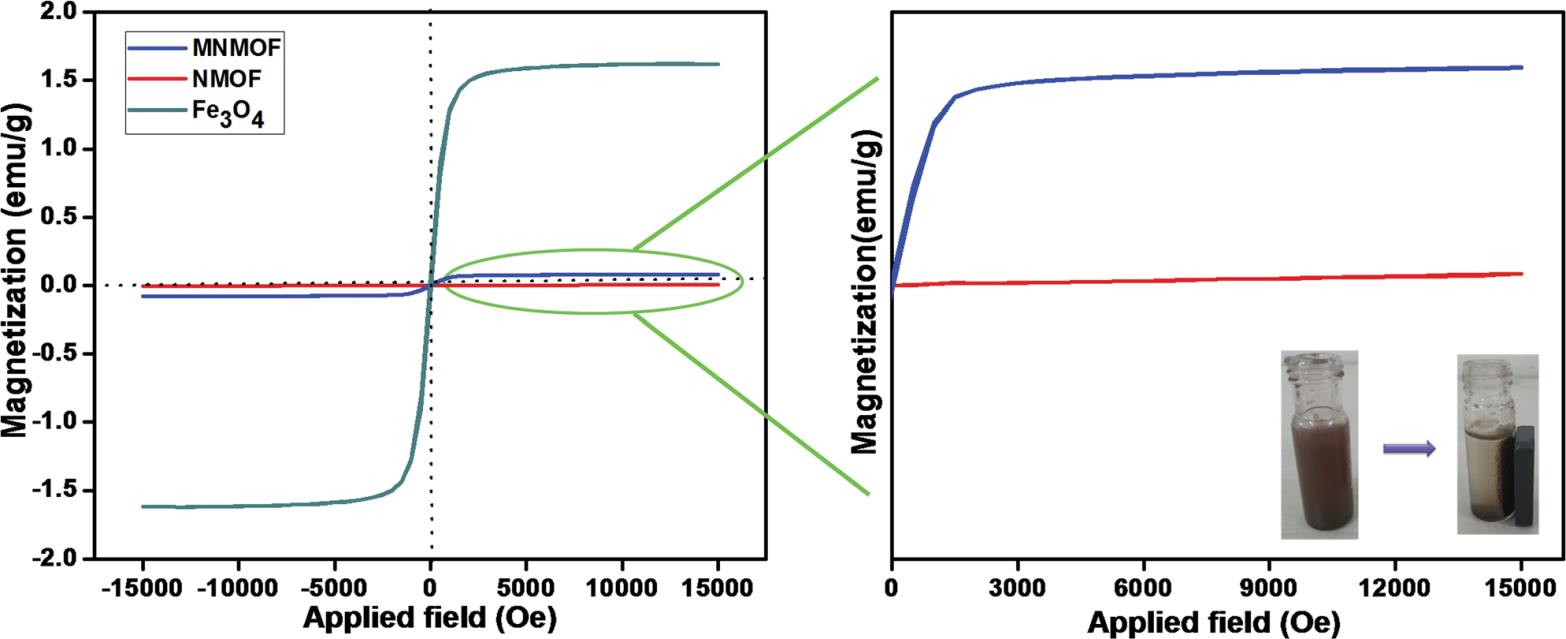
Hysteresis loops of synthesized Fe_3_O_4_ NPs, NMOF and MNMOF. The inset shows digital images for MNMOF before and after magnetic separation achieved using an external magnetic field.

The hysteresis loops and magnetic properties of Fe_3_O_4_ NPs, NMOF and MNMOF were investigated. Fig. 2 (inset) shows that the presence of Fe_3_O_4_ NPs leads to the facile separation of MNMOF from the sensing probe by the application of an external magnet. In this analysis, the MNMOF magnetization curve exhibits a paramagnetic nature (Fig. 2). The pristine Fe_3_O_4_ NPs have a high magnetization value of 1.6 emu compared to that of NMOF (4.2933 × 10^−3^ emu). MNMOF shows a saturation magnetization value that is estimated to be 79.65 × 10^−3^ emu (see the enlarged magnetic curve in Fig. 2), indicating an increase in magnetization and good super magnetism compared to those of NMOF. As shown in the inset to Fig. 2, MNMOF can be easily separated from solution using an externally applied magnet.

**Fig. 3:**
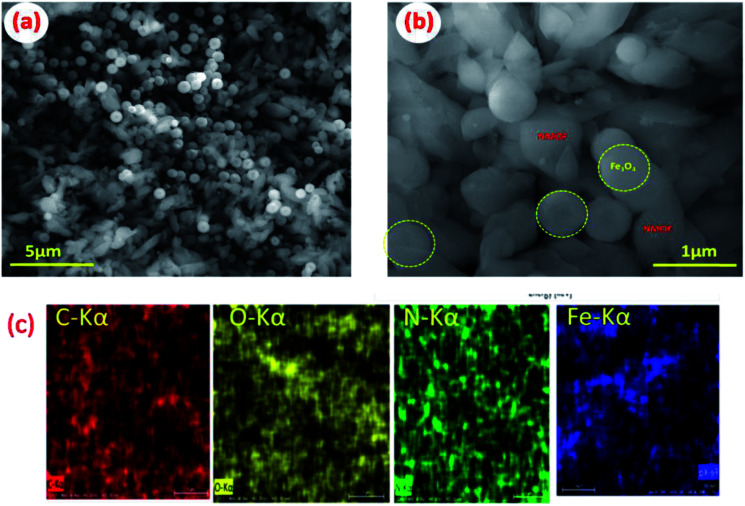
(a and b) SEM images of MNMOF, (c) EDS mapping images of MNMOF.

**Fig. 4:**
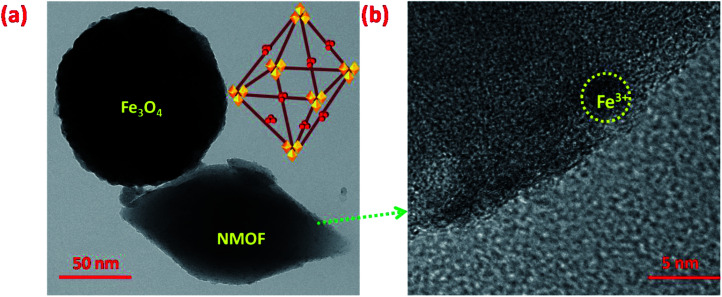
(a) TEM image of MNMOF, (b) HR-TEM image of MNMOF.

Extensive morphological studies were conducted to better understand the synthesized Fe_3_O_4_ NPs, NMOF and MNMOF as shown in Fig. 3, S1, S2, and S9.† Fig. S1† shows that the pristine Fe_3_O_4_ NPs exist as spheres with uniform dispersion. NMOF (MIL-101-NH_2_) displays hexagonal rod-like structures (Fig. S2†), and its surface is smooth with no apparent isolated particles. Fig. 3a and b show SEM images which confirm the formation of MNMOF. It is constructed from Fe_3_O_4_ NPs and NMOF and the particles are uniformly dispersed. In addition, energy-dispersive X-ray spectroscopy (EDX) analysis indicates that MNMOF consists of Fe, C, O, and N elements, and that Fe_3_O_4_ NPs are anchored on the surface of MNMOF (Fig. S3†). Furthermore, the EDS elemental mapping also confirms that Fe, C, O, and N elements are distributed on the surface of MNMOF (Fig. 3c). Additionally, HR-TEM microscopy images evidently show Fe_3_O_4_ NPs anchored on the surface of MNMOF rods (Fig. S3†). The HR-TEM microscopy images of MNMOF are displayed in Fig. 4. As can be seen in Fig. 4, Fe_3_O_4_ NPs are anchored on the surface of the MNMOF rods with a narrow size distribution.

**Fig. 5:**
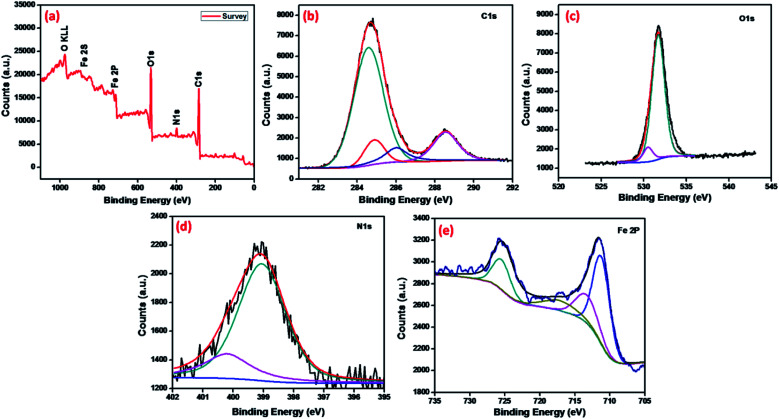
XPS spectra of MNMOF: (a) survey, (b) C 1s, (c) O 1s, (d) N 1s and (e) Fe 2p.

The chemical analysis and bonding configuration of MNMOF were investigated by X-ray photoelectron spectroscopy (XPS). The survey spectrum (Fig. 5a) shows C 1s, N 1s, O 1s, Fe 2p and Fe 2s peaks at 284.9, 399.1, 532.09, 726.5 and 894.46 eV, respectively.^39^Fig. 5b shows the high-resolution XPS spectrum of C 1s, which emphasizes surface components, such as the benzoic and carboxylate groups of organic linkers with binding energies of 284.8 and 289.4 eV, respectively.^40,41^ The O 1s spectrum can be fitted by two peaks at binding energies of around 532.2 and 531.2 eV, which can be attributed to the organic linker of carboxylate groups and Fe–O bonds in the Fe_3_O_4_ NPs (Fig. 5c). Fig. 5e shows the high-resolution XPS spectrum of Fe 2p. The binding energies of 713.5, 725.7 and 718.02 eV are characteristic of Fe^3+^ in MNMOF, while the peaks at binding energies of 711.4 and 726.5 eV are typical for magnetite.^42^

### Quenching–quenching mechanism of MNMOF in the detection of Hg(ii)

**Scheme 1:**
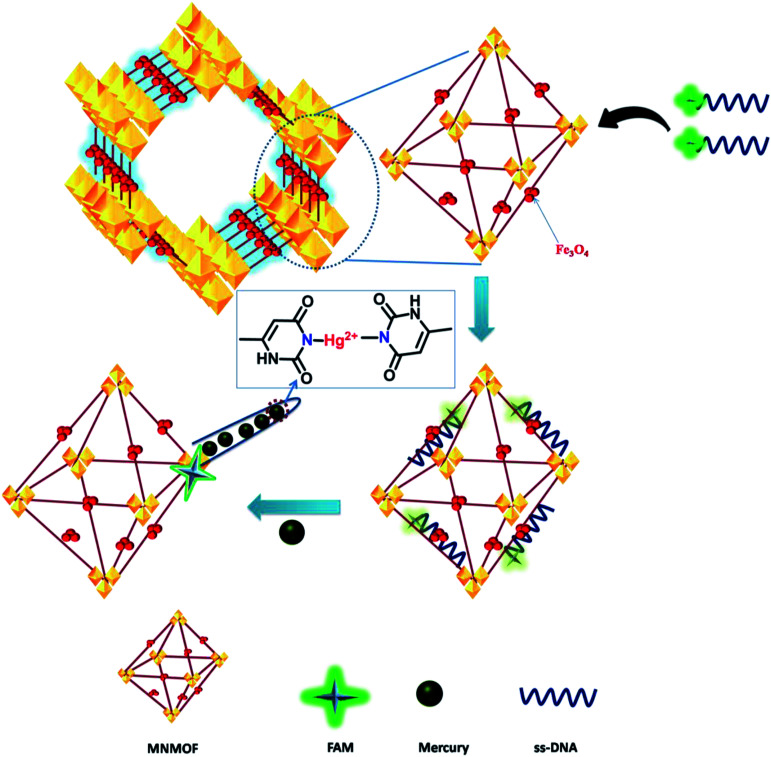
Schematic illustration of the DNA fluorescence assay with MNMOF as a sensing platform.

In general, transition-metal ions have intrinsic fluorescence quenching properties. Recently, very few nanomaterials have been used as fluorescence quenchers, including GO, CNTs, gold NPs and MoS_2_ nanosheets. These nanomaterials have been employed in sensing strategies using fluorescence quenching and recovery of dye-labeled ssDNA. In contrast, MNMOF shows an unusual fluorescence quenching mechanism with FAM-ssDNA. Here, we explored the analytical performance of the novel fluorescence quenching mechanism represented in Scheme 1. FAM-labeled ssDNA is readily adsorbed to the surface of MNMOF *via* non-covalent interactions. The fluorescence of the FAM-labeled ssDNA is partially quenched by this interaction with MNMOF. Then, Hg(ii) ions are introduced to the ssDNA and are hybridized to form a duplex dsDNA. After hybridization, the fluorescence of FAM is further quenched because the negatively charged carboxyl and phenolic hydroxyl groups of FAM-labeled ssDNA interact with Fe^3+^ ions on the surface of MNMOF. Further, FAM-dsDNA does not dissociate away from MNMOF, but re-adsorbs on the surface of Fe^3+^ in MNMOF.

**Fig. 6:**
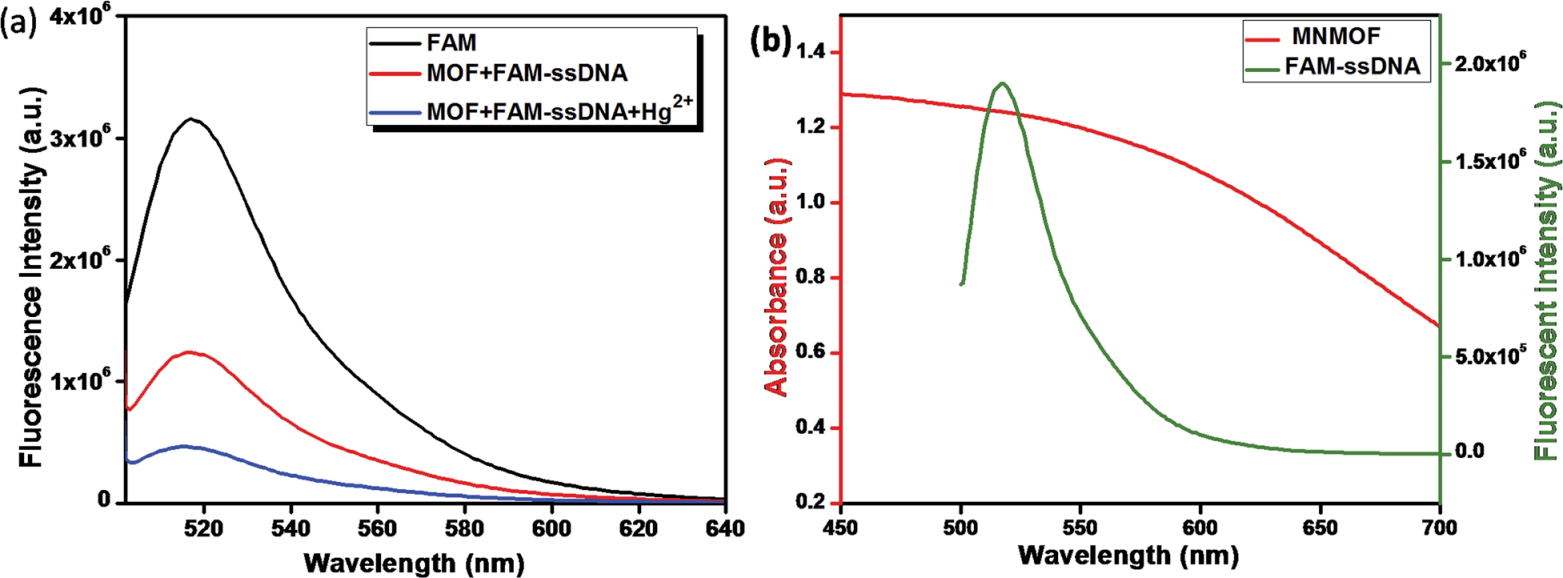
(a) Schematic illustration of the quenching mechanism and (b) fluorescence spectra of FAM, MNMOF + FAM-ssDNA and MNMOF + FAM-ssDNA + Hg(ii).

The fluorescence quenching mechanism was investigated by fluorescence and UV-visible spectroscopy. As shown in Fig. 6a, FAM-ssDNA has obvious fluorescence emission at 520 nm (black line in Fig. 6a). The addition of MNMOF leads to the formation of a hybrid complex that causes partial quenching of the fluorescence intensity (red line in Fig. 6a). Furthermore, the introduction of Hg(ii) ions into the solution containing the hybrid complex results in the further quenching of the fluorescence intensity (blue line in Fig. 6a). The efficient quenching can be attributed to the strong adsorption of FAM-labeled ssDNA on the surface of MNMOF through π–π stacking interactions. The fluorescence intensity is decreased due to FRET as displayed in Fig. 6b.

**Fig. 7:**
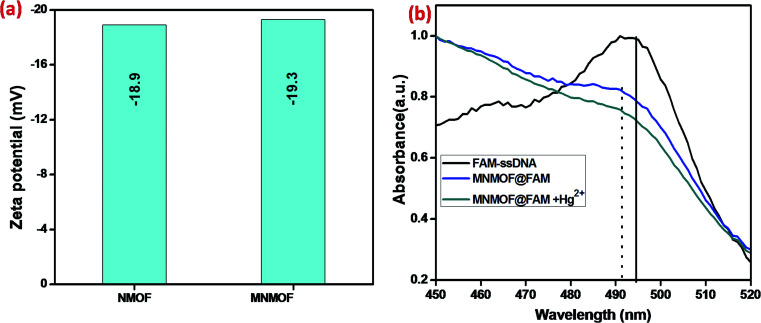
(a) Zeta-potentials of NMOF and MNMOF, (b) UV-visible spectra of FAM-ssDNA, MNMOF@FAM-ssDNA, and MNMOF@FAM-ssDNA + Hg(ii).

Further, the zeta potentials were measured and used to identify the lattice charges of the synthesized materials, as shown in Fig. 7a. We established that NMOF has a negative surface charge. After incorporation of Fe_3_O_4_ NPs to form MNMOF, the negative zeta potential becomes slightly greater. The UV-visible spectra also provide evidence for a unique quenching mechanism; the results are displayed in Fig. 7b. It can be seen that in the absence of MNMOF, FAM-ssDNA shows an obvious fluorescence emission at 495 nm in the Tris–HCl buffer solution. When MNMOF and FAM are combined, the soret band of FAM shifts to a lower wavelength (blue shift). Furthermore, when the Hg(ii) target ions are added into the hybrid complex there is no further shift. As a result, these spectra reveal that FAM-ssDNA does not desorb from the surface of MNMOF when the Hg(ii) ions are added. This is probably since MNMOF contains more uncoordinated Fe^3+^ ions, which bind the dsDNA.

### Fluorescence quenching properties

**Fig. 8:**
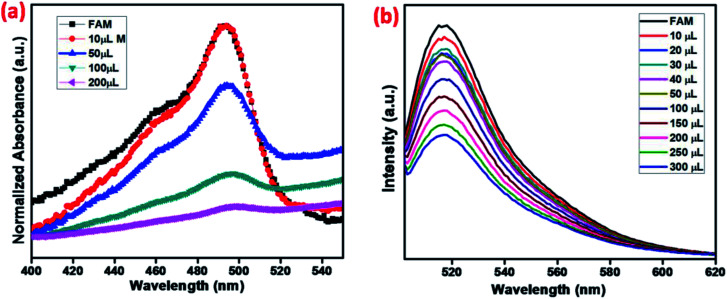
(a) UV-visible spectra and (b) fluorescence spectra showing the quenching properties of FAM-ssDNA with various amounts of MNMOF.

MNMOF has excellent fluorescence quenching properties, as shown in Fig. 8. The UV-visible absorption spectrum of FAM-ssDNA shows an absorption band at 495 nm (Fig. 8a). Upon the addition of MNMOF, that absorption band shows remarkably hypochromicity; we suggest that this is due to a static interaction between MNMOF and the fluorophore of FAM-ssDNA. The fluorophore is conjugated with a plane of MNMOF through π–π interactions and electrostatic interactions. The result is that the absorption band intensity is decreased by MNMOF. The fluorophore of the ssDNA has inherent fluorescence properties and shows a strong emission at 520 nm (Fig. 8b). By adding MNMOF into the solution containing FAM-ssDNA, this fluorescence intensity is also quenched. Moreover, increasing the concentration (50–350 ppm) of MNMOF further quenches the fluorescence intensity, so the fluorescence intensity is inversely proportional to the concentration of MNMOF. Afterwards, we expected the fluorescence intensity to be increased by the introduction of the toxic target ions. In contrast, the intensity of fluorescence emission is quenched even more. In this case, the FAM-ssDNA binds to the target ions to become dsDNA based on T–Hg(ii)–T interactions.

### Optimization and feasibility study

**Fig. 9:**
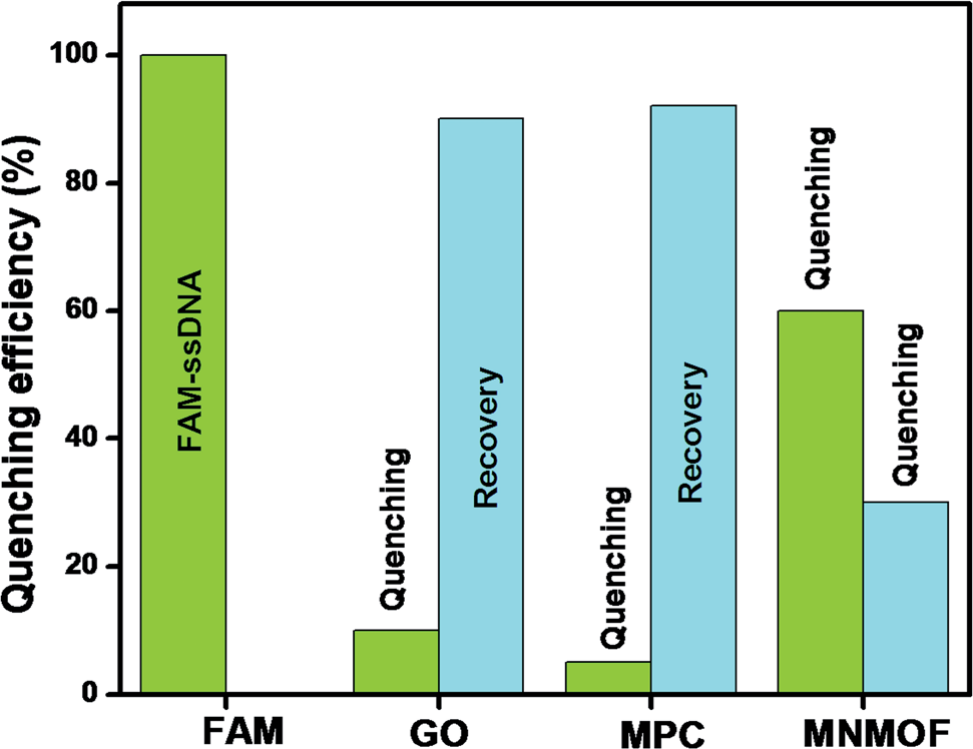
A comparison of GO, MPC, and MNMOF for quenching and recovery of FAM-ssDNA.

To optimize the sensing performance of MNMOF, we investigated several influencing factors such as the concentration of MNMOF, quenching time, recovery time and probe concentration. The concentration of MNMOF was first optimized. As observed in Fig. S3,† the FAM-ssDNA fluorescence intensity gradually decreases with the increase in the concentration of MNMOF. To further understand the adsorption kinetics behavior of the hybridization of FAM-labeled ssDNA and MNMOF, Hg(ii) ions were added during the incubation time (Fig. S5†). In the absence of Hg(ii), the fluorescence signal shows a decrease within 1 minute and it reaches equilibrium at around 7 minutes. The hypochromic curves suggest that MNMOF can adsorb FAM-ssDNA effectively and quickly. After the introduction of Hg(ii) ions, the fluorescence intensity decreases gradually (Fig. S6†) and reaches equilibrium at around 6 minutes, suggesting that Hg(ii) binds to the ssDNA to form duplex structures which are effectively re-adsorbed onto MNMOF in a short time. Different concentrations of the probe were added to MNMOF and the results are displayed in Fig. S7†. Then 10 μL of MNMOF was used with different concentrations of FAM-ssDNA. MNMOF initially decreases the fluorescence intensity but this increases again with increasing concentrations of FAM-ssDNA. The rapid adsorption and re-adsorption of FAM-ssDNA makes MNMOF promising for the fluorescence sensing of Hg(ii) ions. Fig. 9 shows a comparison of MNMOF with other quencher materials (GO, and MOF-derived Fe_3_O_4_ (MPC)) for the quenching and recovery of fluorescence after the addition of the quencher and then Hg(ii). MNMOF exhibits a unique quenching mechanism for the detection of Hg(ii) ions.

### Detection specificity

**Fig. 10:**
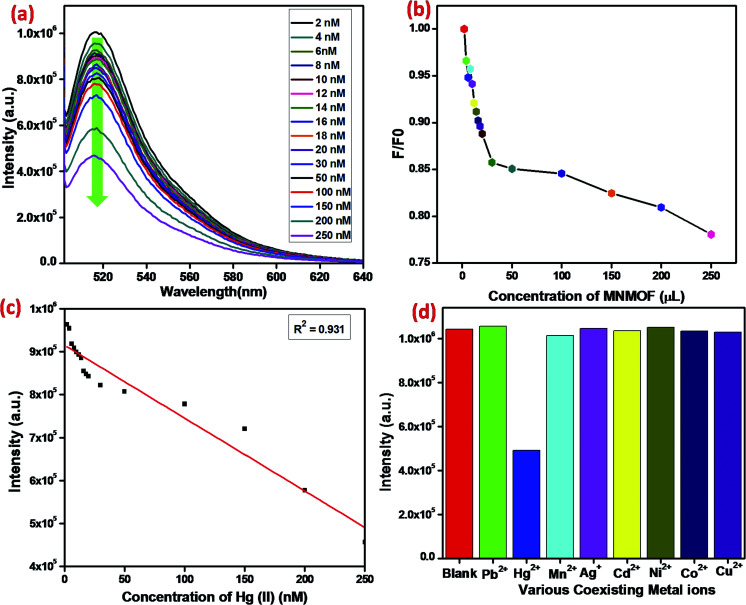
(a) Fluorescence emission spectra of the FAM-labeled ssDNA and MNMOF with different concentrations of Hg(ii), (b) fluorescence intensity verses Hg(ii), concentration, (c) the linear relationship between the fluorescence intensity and Hg(ii) concentration, (D) the selectivity of MNMOF and FAM-ssDNA for Hg(ii) in comparison to that for other co-existing metal ions.

In addition to detection sensitivity, selectivity is a critical factor for evaluating the performance of a proposed sensing system (Fig. 10). Indeed, ssDNA can be used as specific probes in many types of analysis, including for DNA, RNA, protein, small molecules, and other metal ions. The different affinities of MNMOF, ssDNA and their hybrid complex could be used as a universal platform to detect harmful heavy metal ions. Typically, a T-rich ssDNA is designed for the detection of Hg(ii) based on its specific interaction with thymine. When 1 mg mL^−1^ of MNMOF is added to 300 μL of FAM-ssDNA (20 nm), adsorption of the FAM-ssDNA occurs on the surface of MNMOF. Therefore, the fluorescence emission is quenched. Moreover, increasing the concentration (50–350 ppm) of MNMOF further quenches the fluorescence intensity (Fig. 10b). The fluorescence intensity is inversely proportional to the concentration of MNMOF. When Hg(ii) is added, the fluorescence intensity is additionally quenched due to the formation of a duplex structure (T–Hg(ii)–T) involving the thymine N3 position. Fig. 10c shows the linear relationship between the concentration of Hg^2+^ ions (2–20 nm) and the fluorescence intensity; a correlation coefficient value of *R*^2^ = 0.931 is obtained and the detection limit is 8 nM according to the 3σ method. The specificity of the sensing strategy was investigated by testing the fluorescence response to Hg^2+^, when the sensor was challenged with other interfering metal ions including Pb^2+^, Mn^2+^, Ag^+^, Cd^2+^, Ni^2+^, Co^2+^, Cu^2+^. As shown in Fig. 10d, the fluorescence intensity is lower for Hg^2+^ compared with that for interferent ions; these results reveal excellent selectivity for Hg^2+^ ions. Thus, T-rich ssDNA gives specific detection of Hg^2+^ based on its specific interaction with MNMOF.

### Real sample analysis

To explore the practical application of the proposed sensor, it was used for the determination of Hg(ii) in a water sample. The water sample was filtered several times before use. Different concentrations of Hg(ii) (0, 5, 10 and 20 nM) were spiked into the water sample and analyzed; the results are summarized in Table 1. It can be noted that the proposed biosensor shows satisfactory results and recovery values of around 70%. The findings clearly suggest that our sensing system could be successfully applied to the analysis of Hg(ii) in real environmental samples.

**Table 1:** Determination of Hg(ii) in environmental water samples using the proposed method

Spiked (Hg(ii)) (nM)	Found (mean ± SD) (nM)	Detected (nM)	Recovery
0	—	—	—
5	3.5 ± 0.25	3.5	70
10	7.5 ± 0.15	7.5	70.5
20	14 ± 0.05	14	70

## Conclusion

In summary, we describe a nucleic acid-based fluorescent biosensor platform that was constructed using FAM-ssDNA with a magnetic nanoscale metal–organic framework (MNMOF) MNMOF was fabricated by a one-pot synthetic method. This effective fluorescence biosensor could be used for the selective detection of Hg(ii) ions over other co-existing metal ions. Non-covalent interactions were employed to adsorb FAM-labeled ssDNA onto the surface of MNMOF and the fluorescence emission was partially quenched. Subsequently, when the Hg(ii) ions were introduced, the fluorescence emission was quenched further at 52%, due to the presence of uncoordinated Fe^3+^ ions on the surface of MNMOF which re-adsorbed the dsDNA formed after hybridization with Hg(ii). The proposed quenching–quenching approach of the described sensor for Hg(ii) gave a good linear response (*R*^2^ = 0.934) and a detection limit of 8 nM which compare well to the performance of previously reported sensors. MNMOF has been shown to be an excellent sensing platform, which will stimulate more exciting research; it can potentially be applied to various other heavy metals and biomolecules.

## Conflicts of interest

There are no conflicts to declare.

## Supplementary Material

RA-010-C9RA08274C-s001

## References

[cit1] Lubick N., Malakoff D. (2013). Science.

[cit2] U.S. EPA , National Primary Drinking Water Regulations, https://www.epa.gov/ground-water-and-drinking-water/national-primary-drinking-water-regulations, accessed Mar 10, 2018

[cit3] Lin Z. H., Zhu G., Zhou Y. S., Yang Y., Bai P., Chen J., Wang Z. L. (2013). Angew. Chem., Int. Ed..

[cit4] Pu Q., Sun Q. (1998). Analyst.

[cit5] Li Y., Chen C., Li B., Sun J., Wang J., Gao Y., Zhao Z., Chai Z. (2006). J. Anal. At. Spectrom..

[cit6] Krachler M., Mohl C., Emons H., Shotyk W. (2002). Spectrochim. Acta, Part B.

[cit7] Aranda P. R., Pacheco P. H., Olsina R. A., Martinez L. D., Gil R. A. (2009). J. Anal. At. Spectrom..

[cit8] Yang J., Foley R., Low G. K. C. (2002). Analyst.

[cit9] Ravikumar A., Panneerselvam P., Radhakrishnan K. (2018). Microchim. Acta.

[cit10] Saran R., Liu J. (2016). Anal. Chem..

[cit11] Singh S., Mitra K., Shukla A., Singh R., Gundampati R. K., Misra N., Maiti P., Ray B. (2017). Anal. Chem..

[cit12] Li B., Du Y., Dong S. (2009). Anal. Chim. Acta.

[cit13] Gao L., Lian C., Zhou Y., Yan L., Li Q., Zhang C., Chen L., Chen K. (2014). Biosens. Bioelectron..

[cit14] Sudibya H. G., He Q., Zhang H., Chen P. (2011). ACS Nano.

[cit15] Huang C. C., Yang Z., Lee K. H., Chang H. T. (2007). Angew. Chem..

[cit16] Libing Z., Tao L., Bingling L., Jing L., Erkang W. (2010). Chem. Commun..

[cit17] Ravikumar A., Panneerselvam P., Radhakrishnan K., Anand Babu C. A., Sivanesan S. (2018). Appl. Surf. Sci..

[cit18] Ravikumar A., Panneerselvam P. (2018). Analyst.

[cit19] He C., Liu D., Lin W. (2015). Chem. Rev..

[cit20] Fang J. M., Leng F., Zhao X. J., Hu X. L., Li Y. F. (2014). Analyst.

[cit21] Karthick P., Pandikumar A., Preeyanghaa M., Kowsalya M., Neppolian B. (2017). Microchim. Acta.

[cit22] William P. L., Soumya M., Nathan D. R., Aamod V. D., Jing L., Sujit K. G. (2017). Chem. Soc. Rev..

[cit23] Morris W., Briley W. E., Auyeung E., Cabezas M. D., Mirkin C. A. (2014). J. Am. Chem. Soc..

[cit24] Della R. J., Liu D., Lin W. (2011). Acc. Chem. Res..

[cit25] Li Y. A., Zhao C. W., Zhu N. X., Liu Q. K., Chen G. J., Liu J. B., Zhao X. D., Ma J. P., Zhang S., Dong Y. B. (2015). Chem. Commun..

[cit26] Wei X., Zheng L., Luo F., Lin Z., Guo L., Qiu B., Chen G. (2013). J. Mater. Chem. B.

[cit27] Zhang H. T., Zhang J. W., Huang G., Du Z. Y., Jiang L. H. (2014). Chem. Commun..

[cit28] Guo J. F., Fang R. M., Huang C. Z., Li Y. F. (2015). RSC Adv..

[cit29] Tian J., Liu Q., Shi J., Hu J., Asiri A. M., Sun X., He Y. (2015). Biosens. Bioelectron..

[cit30] Ravikumar A., Panneerselvam P., Morad N. (2018). ACS Appl. Mater. Interfaces.

[cit31] Marieeswaran M., Panneerselvam P., Ravikumar A., Sivanesan S. (2019). Analyst.

[cit32] Sijia L., Jianan C., Xia W., Xuan Z., Qi H., Xiaohong H. (2019). J. Hazard. Mater..

[cit33] Yuling H., Zelin H., Jia L., Gongke L. (2013). Anal. Chem..

[cit34] Huijun L., Qingqing L., Xinglei H., Ning Z., Zhouqing X., Yan W., Yuan W. (2018). Cryst. Growth Des..

[cit35] Caihong Z., Lunhong A., Jing J. (2015). J. Mater. Chem. A.

[cit36] Anand Babu Christus A., Panneerselvam P., Ravikumar A. (2018). Anal. Methods.

[cit37] Zhiguang Z., Xinyong L., Baojun L., Qidong Z., Guohua C. (2016). RSC Adv..

[cit38] Ai L., Zhang C., Liao F., Wang Y., Li M., Meng L., Jiang J. (2011). J. Hazard. Mater..

[cit39] Tama S. K., Dusseault J., Polizu S., Menard M., Halle J.-P., Yahia L. H. (2005). Biomater.

[cit40] Wang N., Zhu L., Wang D., Wang M., Lin Z., Tang H. (2010). Ultrason. Sonochem..

[cit41] Zhao H., Wang Y., Wang Y., Cao T., Zhao G. (2012). Appl. Catal., B.

[cit42] Su J., Cao M., Ren L., Hu C. (2011). J. Phys. Chem. C.

