# Supplementary material for: A magnetic nanoscale metal–organic framework (MNMOF) as a viable fluorescence quencher material for ssDNA and for the detection of mercury ions via a novel quenching–quenching mechanism
Source: RSC Adv. 2020 Jan 22;10(7):3705–14. doi: 10.1039/c9ra08274c (PMC9048829; doi:10.1039/c9ra08274c)
Supplement: RA-010-C9RA08274C-s001 [file RA-010-C9RA08274C-s001.pdf]

Electronic Supplementary Material (ESI) for RSC Advances.
This journal is © The Royal Society of Chemistry 2020

Electronic supporting information

Magnetize Nanoscale Metal-Organic Framework (MNMOFs) as a viable fluorescent quencher material for ssDNA based on a Novel Quenching-Quenching mechanism for detection of mercury ions

Muppidathi Marieeswaran, Perumal Panneerselvam *

* Department of Chemistry, SRM Institute of Science and Technology, Kattankulathur

603 203, Tamil Nadu, India

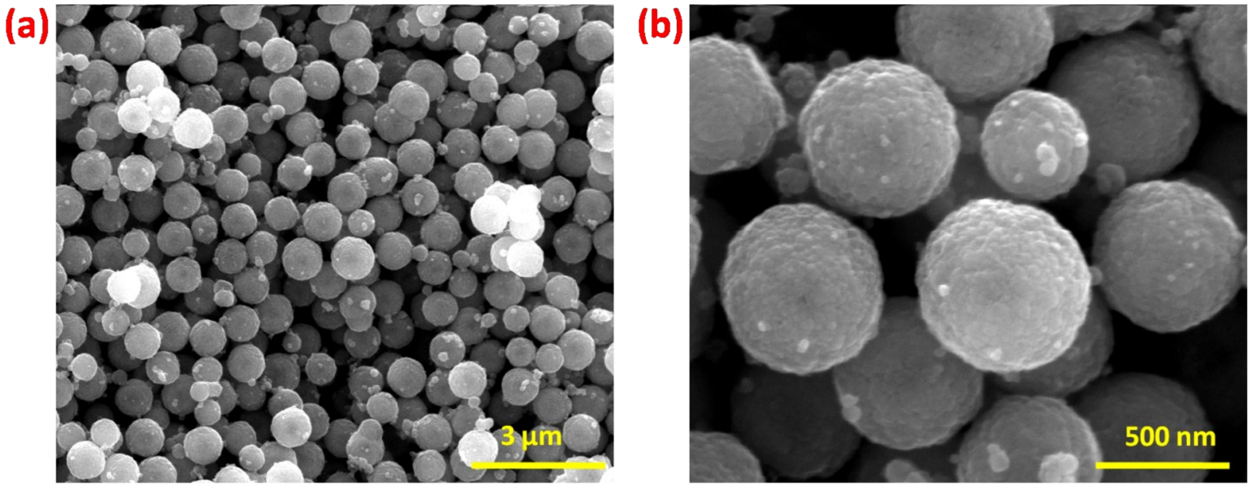

Fig.S1 (a,b) SEM Images Fe_3O_4 NPs

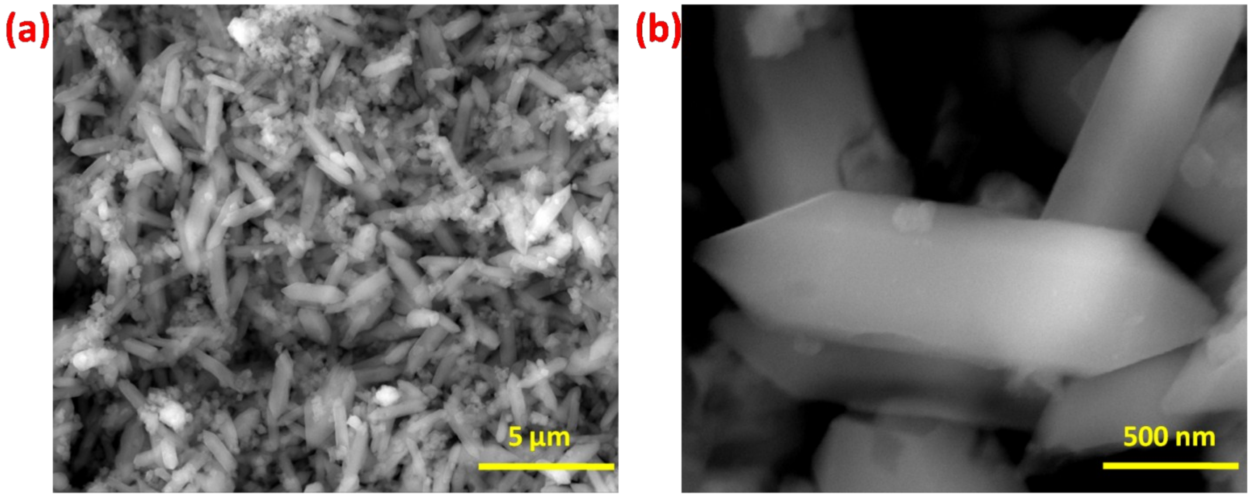

Fig.S2 (a,b) SEM Images MIL-101-NH_2 by hydrothermal method synthesis method

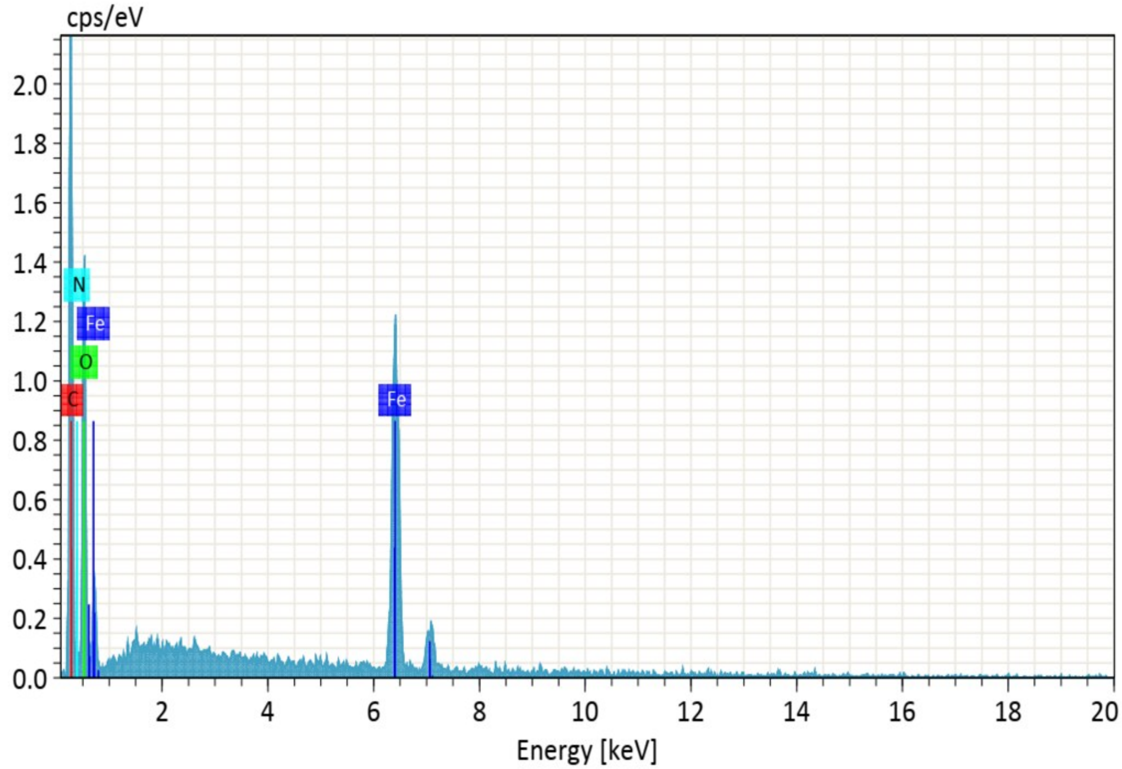

Fig.S3 EDX spectra of MNMOF

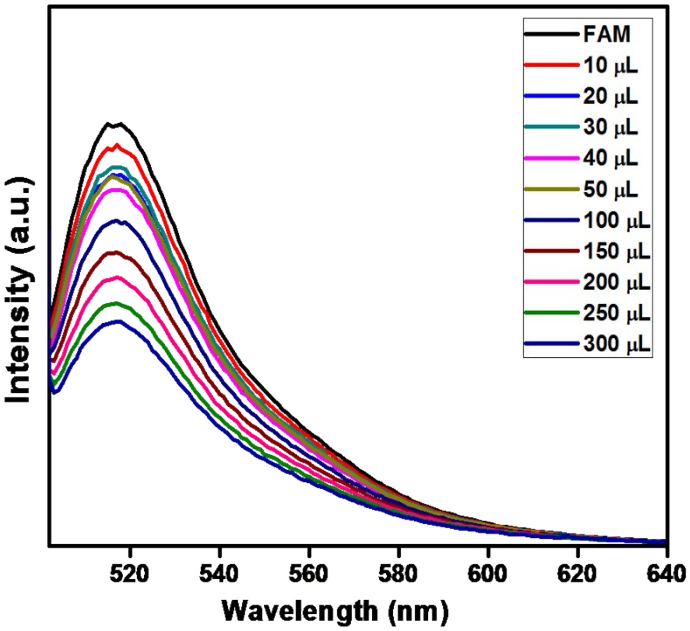

Fig.S4 Different concentration of NMOFs with FAM-ssDNA

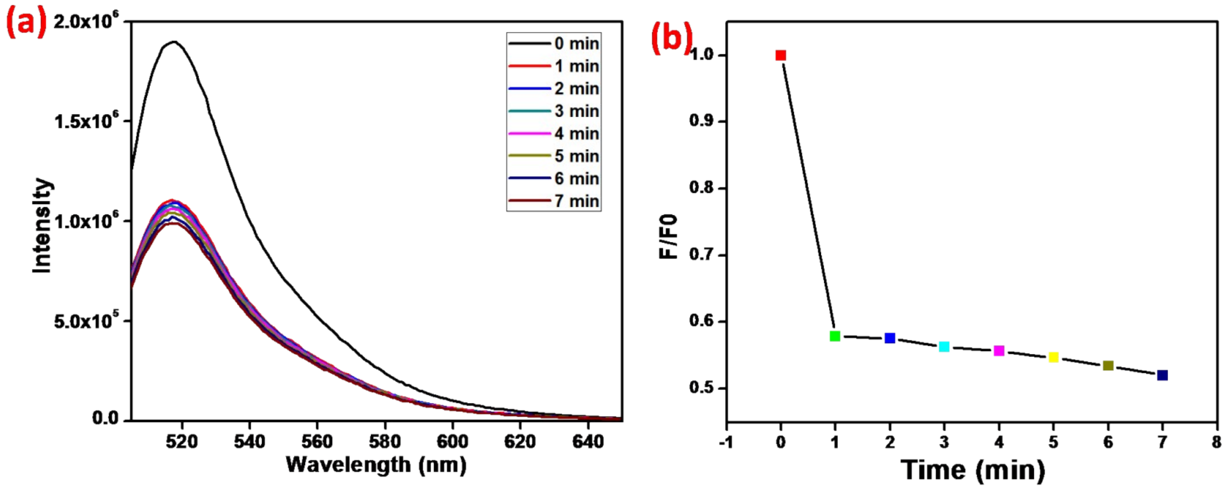

Fig.S5. Time dependent fluorescent quenching of FAM-ssDNA

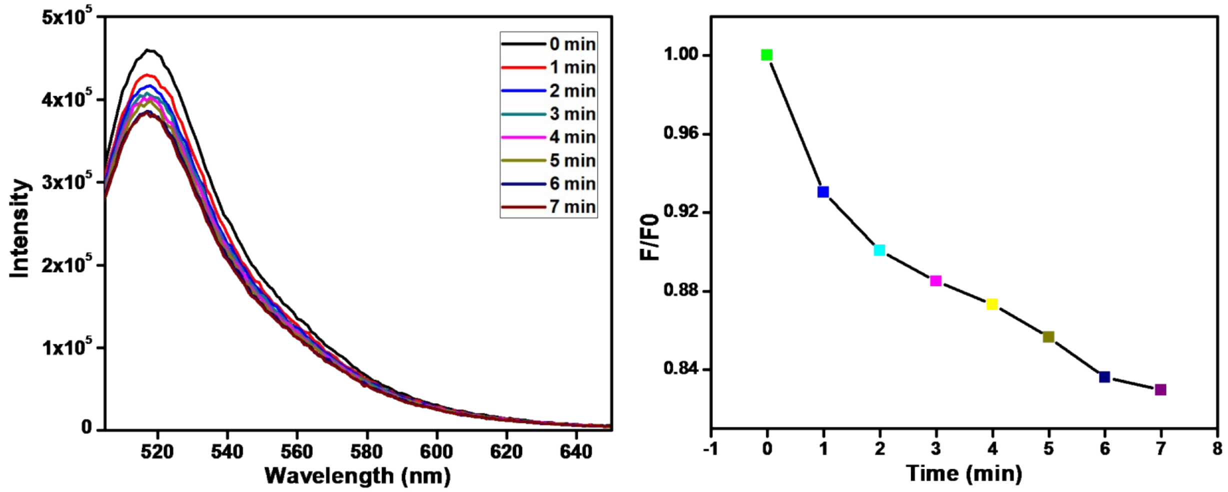

Fig.S6 Time dependent fluorescent quenching of Hg^{2+}FAM-ssDNA

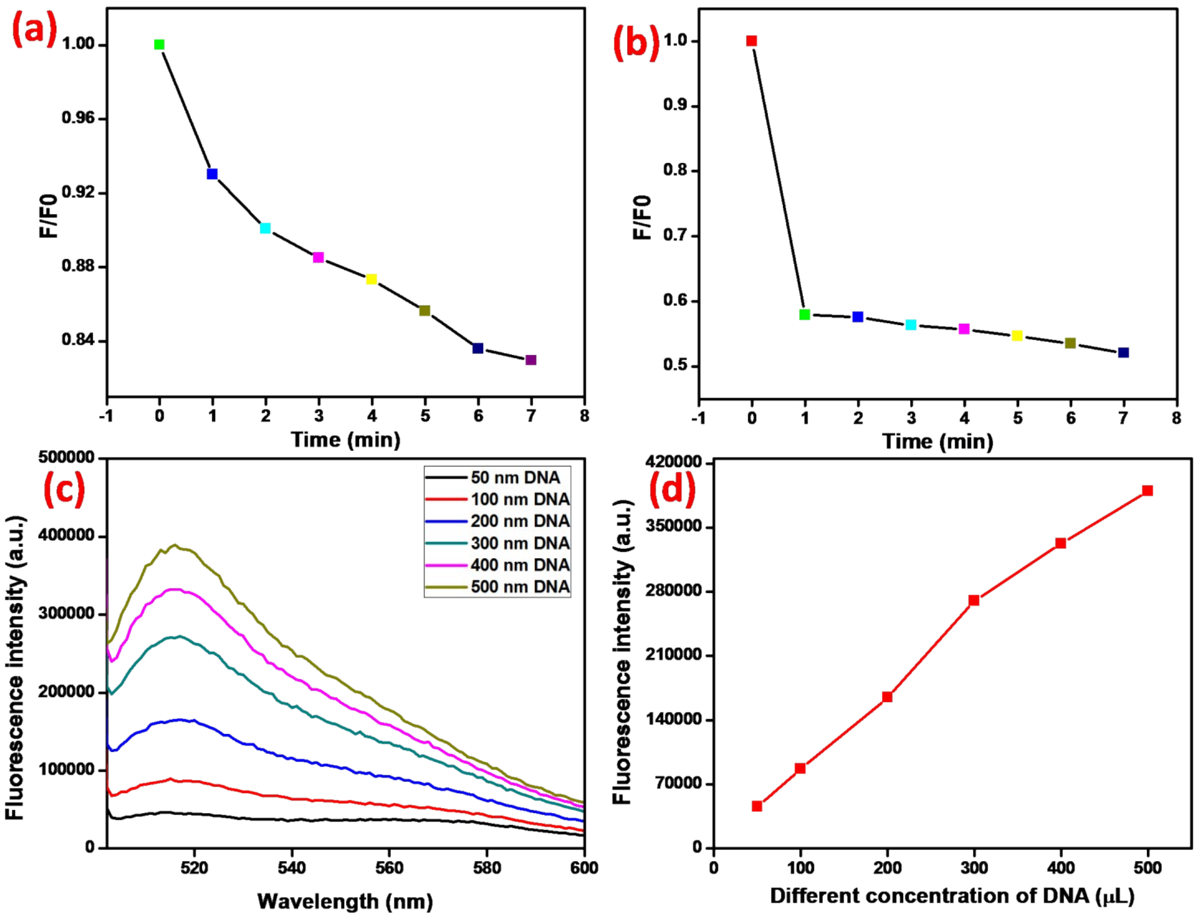

Fig.S7 (a) adsorption of FAM-ssDNA and MNMOF, (b) adsorption of FAM-ssDNA and MNMOF+Hg^{2+}, (c) different concentration of FAM-ssDNA into 1wt% of MNMOF, (d) the relative fluorescence intensity detection of adsorption of FAM-ssDNA with MNMOF.

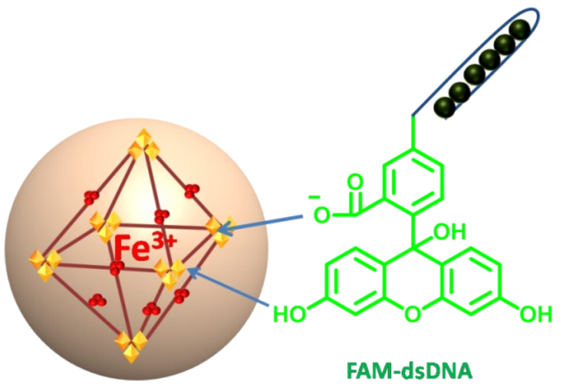

Fig.S8 illustration of FAM-ssDNA+Hg^{2+} with MNMOF.

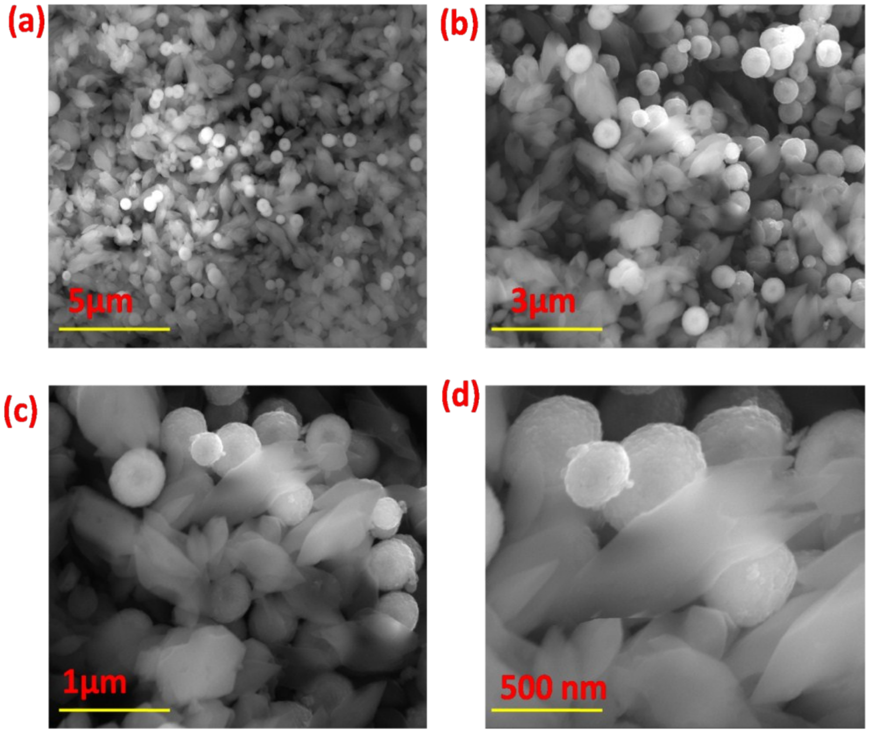

Fig. S9 SEM images with various magnitudes
